# The role of conflict processing in multisensory perception: behavioural and electroencephalography evidence

**DOI:** 10.1098/rstb.2022.0346

**Published:** 2023-09-25

**Authors:** Adrià Marly, Arek Yazdjian, Salvador Soto-Faraco

**Affiliations:** ^1^ Center for Brain and Cognition, Universitat Pompeu Fabra, 08005 Barcelona, Spain; ^2^ Institució Catalana de Recerca i Estudis Avançats, 08010 Barcelona, Spain

**Keywords:** conflict monitoring, causal inference, reaction time, ventriloquist illusion, EEG, intersensory conflict

## Abstract

To form coherent multisensory perceptual representations, the brain must solve a causal inference problem: to decide if two sensory cues originated from the same event and should be combined, or if they came from different events and should be processed independently. According to current models of multisensory integration, during this process, the integrated (common cause) and segregated (different causes) internal perceptual models are entertained. In the present study, we propose that the causal inference process involves competition between these alternative perceptual models that engages the brain mechanisms of conflict processing. To test this hypothesis, we conducted two experiments, measuring reaction times (RTs) and electroencephalography, using an audiovisual ventriloquist illusion paradigm with varying degrees of intersensory disparities. Consistent with our hypotheses, incongruent trials led to slower RTs and higher fronto-medial theta power, both indicative of conflict. We also predicted that intermediate disparities would yield slower RTs and higher theta power when compared to congruent stimuli and to large disparities, owing to the steeper competition between causal models. Although this prediction was only validated in the RT study, both experiments displayed the anticipated trend. In conclusion, our findings suggest a potential involvement of the conflict mechanisms in multisensory integration of spatial information.

This article is part of the theme issue ‘Decision and control processes in multisensory perception’.

## 1. Introduction

To form coherent perceptual representations, the brain must combine sensory cues deriving from the same event, but segregate cues that refer to different events. However, information about which cues arise from a common cause and which are independent is uncertain and must be inferred. To do so, the perceptual system must solve the so-called causal inference problem [1,2]: deciding whether two (or more) sensory cues originated from the same event and, therefore, should be integrated in a unitary perceptual representation or, else, they have different origins and should be processed independently (i.e. segregated). This perceptual decision process regarding the causal origin of multiple sensory stimuli has motivated extensive empirical work and theoretical proposals, from the unity assumption of the 1980s [3] to current approaches of Bayesian causal inference [1,2]. This latter approach describes the process as a combination of current sensory evidence (i.e. the likelihood) with prior knowledge. Here, we address the hypothesis that the conflict monitoring mechanisms, from the cognitive control network, play a substantial role in this causal attribution during multisensory perception.

**Figure 1.**
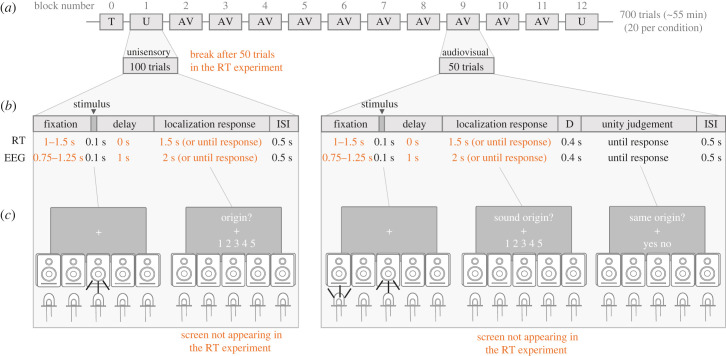
Schematic representation of the experimental design. Highlighted in orange are the differences between the reaction times (RT) and the electroencephalography (EEG) experiment. (*a*) Organization of the entire experiment's blocks. Participants started with a training block (T), followed by a unisensory block (U), 10 audiovisual blocks (AV) and a final unisensory block (U). In the RT experiment, participants could rest in the middle of the unisensory block (after 50 trials). (*b*) Trial structure in the unisensory and audiovisual blocks. The fixation duration for the RT experiment was longer (randomly chosen between 1 and 1.5 s) than the EEG protocol (0.75 to 1.25 s), and speeded localization responses were accepted from right after the stimulus until a 1.5 s response deadline. In the EEG experiment, to prevent conflating response preparation with induced activity, there was a delay period of 1 s before the participant could answer to the location of the sound (with an additional 2 s response deadline). (*c*) Examples of stimuli presentation and the response screens for the unisensory and audiovisual trials. In the RT experiment, the localization response screen did not appear (as participants could answer right after stimulus presentation).

In the Bayesian framework, the brain has access to noisy sensory evidence from cues in each modality, and the disparity between the cues (also referred to as sensory conflict or intersensory discrepancy [3,4]) plays a crucial role in the causal inference process. The smaller the disparity, the bigger the preference for an integration (or fusion) model, whereas larger disparity favours the segregation model (see [5] for a review). This process can be studied, for example, with the well-known ventriloquist illusion paradigm, where the perceived location of a sound is biased towards the location of a synchronized visual event occurring nearby [2]. In this paradigm, light and sound are presented from a range of locations (figure 1), and participants have to locate either one or both stimuli. Sound location is often ‘captured’ by the light (i.e. the sound is perceived as coming from, or near, the position of the light) when spatial disparity is small.

We propose that during the causal inference process, the arbitration between alternative causal models (representing the integration or segregation of the stimuli), involves competition that engages the brain mechanisms of conflict processing. If our hypothesis is true, a conflict signal similar to the one seen in other conflict paradigms would be triggered in the brain. The theory of conflict monitoring and signalling, described in Botvinick *et al*. [6] and further developed more recently, established the anterior cingulate cortex (ACC) as a relevant area in conflict monitoring and detection. At present, there is evidence that conflict processing involves an extended network of brain areas (including dorsolateral prefrontal cortex, inferior frontal gyrus), and that the ACC is associated with a variety of processes in addition to conflict monitoring [7], such as error processing [8], task effort prediction [9], reward prediction [10] or pain perception [11]. It is likely that the ACC has a much more sophisticated computational function than just monitoring for conflict or signalling errors. For example, it has been suggested that its function involves anticipating a cost–benefit signal for the potential reward value of planned actions (see [12] for a review), or that it has a role in initiating the mechanisms that motivate the execution of high-level plans [13]. Despite that, the ACC involvement in classical conflict paradigms, such as the Stroop [14,15], the Flanker [16] or the Simon [17,18] tasks, as well as in internal sampling during perception [19], has been demonstrated. In these classic conflict tasks, congruent trials are contrasted with incongruent trials where two stimuli (or features within a stimulus) point to different answers creating a motor conflict that must be overridden. The reaction time (RT) as well as the ACC activity increase in incongruent trials compared to congruent trials. This ACC activation has been observed in functional magnetic resonance imaging (fMRI) [17] and electroencephalography (EEG) studies [18]. With EEG, ACC activity is inferred from differences in event-related potentials (ERPs) [20] and in the theta band activity from fronto-medial (fm) electrodes [18].

The involvement of conflict detection and resolution mechanisms during intersensory conflict remains largely unknown. Most of the existing evidence regarding ACC activity during conflict is based on motor conflict situations, and studies investigating similar conflict responses in stimulus-stimulus conflict are scarce (but see [21]). It is important to note that the initial theoretical proposal of the cognitive control theory explicitly mentioned the extension of the theory to stimulus–stimulus conflict; however, this prediction has only started to be explored recently. Some of the initial studies addressing this used intersensory conflict [22–24]. These studies reported increased activation of the ACC in response to McGurk syllables (i.e. syllables where the auditory percept is modified by the sight of incongruent lip movements) compared to congruent audiovisual syllables using fMRI [23]. A separate study, also using McGurk syllables, reached similar conclusions with EEG measurements of fm-theta [22].

We could find three more studies that examined/reported ACC activity in multisensory paradigms, with varying interpretations. In an fMRI study using a ventriloquist paradigm with three disparities, increased ACC activity was observed for audiovisual incongruent trials compared to congruent trials, along with activations in other regions [25]. However, for the functional imaging results, they did not distinguish between the different degrees of disparity. Another experiment that used a task which involved a mixture of motor and stimulus conflict regarding seen letters and heard letter names, reported an increase in RT as well as in the ACC activity for incongruent compared to congruent trials [26]. However, this study could be said to fall in, or be indistinguishable from, a response conflict type of protocol. Finally, an fMRI study investigating cross-modal semantic conflict reported increased ACC activity for semantically congruent pairs of images and sounds compared to incongruent pairs [27]. To our knowledge, this is the only study that identified ACC activity to increase for the congruent case, and it has not been replicated in other cross-modal semantic congruence fMRI studies (see [28,29] for reviews). Methodological differences, such as presenting congruent or incongruent stimuli in blocks, may have contributed to the distinct findings in this study, potentially by adaptation effects.

By contrast to multisensory research, visual research has often explicitly addressed the competition processes during perception, in particular regarding bistable stimuli. For example, Weilnhammer *et al*. [30] proposed that competition between alternative representations of ambiguous stimuli engaged the inferior frontal cortex (a region with functional connection to the ACC [31]), as a main player in attributing between the two perceptual interpretations. This proposal is also supported by invasive recordings in primate studies [32]. Another study by Drew *et al*. [33] also addressed visual competition using binocular rivalry, and found fm-theta increases (consistent with ACC activation) prior to impending perceptual switches between the two rival images, with a decrease afterwards. These results are also supported by invasive recordings in the medial temporal and frontal lobes in humans [34]. In both cases, the interpretation is similar to the one we propose for the multisensory case, with the competition between the two alternative perceptual interpretations, or their corresponding neural representations, engaging the conflict processing network.

Based on the mounting evidence that the conflict network might be engaged by incompatible representations of sensory cues, we hypothesize that the conflict monitoring system might be involved more generally during multisensory processing, playing a role in the attribution of causal models during the perceptual inference process. In particular, we propose that the choice of the generative model (or the combination thereof) leads to a competitive process between the alternative representations of the cross-modal events. Also, that this competition between causal models leads to conflict and hence to the potential engagement of the conflict network.

To test this proposal, we designed two experiments based on a ventriloquist illusion paradigm (adapted from Körding *et al*. [2]), that allowed us to address if the conflict monitoring system is engaged differentially in moments of high uncertainty regarding whether audiovisual cues should be integrated or segregated. In each trial, a sound and a light appeared randomly from one of five locations, leading to cross-modal disparities ranging from −24° to 24°. Participants were asked to first locate the sound (localization judgement) and then, to report whether they perceived the two stimuli as coming from the same or different positions (unity judgement). One experiment measured speeded RTs to study the changes in response latencies as a function of disparity, and another experiment measured EEG to study fm-theta power variations as a function of disparity. Both measures are classical markers of conflict. The hypotheses and methods were pre-registered for both experiments (RT: https://osf.io/3sjch, EEG: https://osf.io/yb5wm). This general hypothesis, which has not yet been entertained systematically, leads to two main predictions. A first, more general prediction, is that in audiovisual congruent stimuli (i.e. when sound and light come from the same location), responses will be faster and the theta activity lower compared to incongruent stimuli; similar to what has been observed in motor conflict paradigms. Interestingly, although the ventriloquist phenomenon has been studied experimentally for more than a century [35], ventriloquist paradigms have seldom measured speeded responses. To the best of our knowledge, only one recent study [36] did so but used only congruent versus incongruent trials without disparity variations. According to this study, RTs to incongruent audiovisual trials were indeed slower than to congruent trials, one immediate prediction of our hypothesis. The second, more specific prediction is that at intermediate intersensory disparities conflict will be higher than at large disparities or at zero disparity, since at intermediate disparities there will be more uncertainty about whether the two stimuli should be integrated or segregated. That is RTs and fm-theta power should increase at intermediate disparities and decrease at higher disparities.

## 2. Methods

### (a) Experimental design

The experimental design was almost identical in the RT and the EEG experiments, and therefore here we describe both and only note the differences between them. Visual and auditory stimuli were presented from five different positions in a horizontal semicircle at eccentricities −12°, −6°, 0°, 6° and 12° (zero denoting straight ahead, and negative denoting to the left of the midline). There were 35 different types of trials: 10 unisensory (two stimulus modalities × five locations) and 25 audiovisual (five × five locations). Each type of trial was presented 20 times during the experiment, for a total of 700 trials divided into 12 blocks. The first and the last blocks of the experiment were unisensory (figure 1*a*), where either one light or one sound was presented at each trial, with modality and position randomly chosen on a trial by trial basis. Each unisensory block consisted of 100 trials (two modalities × five locations × 10 repetitions). In the RT experiment, participants could rest in the middle of the block (after 50 trials). The 10 blocks in between were audiovisual (figure 1*a*), with a light and a sound presented synchronously. Each block consisted of 50 trials (each of the 25 possible modality by position combinations were presented twice). Prior to the experiment, participants ran a training block consisting of examples of both types of blocks. Training was repeated until participants could correctly locate most of the unimodal trials (≥ 80%) and were not missing responses in the audiovisual trials.

The structure of each trial was similar in unisensory and audiovisual blocks (figure 1*b*). A central fixation cross (0°) was present throughout the trial, except for the inter-trial interval. The fixation period, jittered randomly between 1000–1500 ms for the RT experiment, and 750–1250 ms for the EEG experiment, was followed by the stimulus presentation (100 ms). In the RT experiment participants were asked to respond right after the stimulus presentation and, therefore, there was no response prompt on the screen. In the EEG experiment participants had to wait for 1000 ms (delay period), until a response prompt appeared on the screen, before giving a response. This was done in order to avoid motor response contamination in the window of interest for the EEG analysis (figure 1*c*). In the unisensory blocks participants answered to the location of the presented stimulus (regardless of its modality). In the audiovisual blocks, participants answered to the location of the sound first, and then to whether or not the sound and the light originated from the same location. Participants had a 1.5 s response deadline starting immediately after the stimuli for the location response in the RT experiment, while the deadline was 2 s for the EEG experiment, starting after the delay period. Unanswered trials were reintroduced later in the same block, up to a maximum of 35 times throughout the experiment. In the RT experiment, participants were instructed to answer as fast and accurately as possible, whereas in the EEG experiment participants were asked to wait until the response prompt appeared, and then respond as accurately as possible. In the 10 audiovisual blocks, the unity judgement question prompt appeared 400 ms after the sound location response, and had no time limit. After the last response in each trial the fixation cross disappeared for 500 ms and the following trial started.

### (b) Participants

The study was approved by the Institutional Committee for Ethical Review of Projects (CIREP) at the UPF. Participants gave their written consent prior to the experiment. A total of 125 participants took part in at least one of the studies (10 of them in both), and received monetary compensation (10€/h^−1^).

Participants were between the age of 18 to 35, right-handed, with normal or corrected to normal vision and audition. Taking drugs that could influence the state of consciousness and/or concentration, having a history of neurological disorders or head injuries, and the inability to understand the instructions in English or Spanish (all by self-report) were causes for exclusion. Participants' data were discarded if they missed more than 5% of the trials throughout the experiment, if the mean absolute error of their responses was greater than 4.5° in any position and modality in the unimodal trials, and/or if the mean absolute error was greater than 3.5° for either modality in the unimodal trials. From the 76 participants recruited in the RT experiment, 55 passed the pre-registered criteria (three could not finish the experiment, seven were excluded for missed trials and 11 for exceeding the mean absolute error of 4.5° in one or more positions). The mean age of the included participants was 22.44 years ± 3.12 (24 males, 31 females). From the 59 participants recruited in the EEG experiment 49 passed the pre-registered criteria (one was excluded for missed trials, eight for exceeding the mean absolute error of 4.5° in one or more positions, and one for having a mean absolute error greater than 3.5° for the mean of the auditory trials). The mean age of the included participants was 23.29 years ± 3.16 (24 males, 25 females).

### (c) Apparatus and stimuli

The protocol was run inside a sound and light attenuated room. The experiment was designed on Python 3.8 [37] using PsychoPy (v2021.2.3) [38] modules: sound, gui, visual, core, data, parallel and keyboard (from hardware); on a computer running Windows 10. Visual stimuli were presented from green LEDs, 100 ms in duration, placed at five different locations arranged in a semicircle (−12°, −6°, 0°, +6° and +12°) centred approximately 68 cm away from the participant's head. The auditory stimuli consisted of pure tones (frequency selected randomly at each trial, from 400 Hz to 480 Hz), presented through five individual speakers (Creative Inspire T6160) placed at the same five locations and distance as the LEDs, and 100 ms in duration. A computer monitor was placed behind the speakers and LEDs montage, to show the instructions, the response prompts, and the fixation cross. Text appeared in grey on a black background.

Participants answered by keypresses through a standard QWERTY keyboard. Location responses were given with fingers 5 to 1 (little finger to thumb) of the left hand, placed on the A, S, D, F, V keys (corresponding to stimulus locations, left-to-right). For unity judgements participants used the index and middle or ring fingers of the right hand, placed on the left and right arrows of the keyboard (corresponding to the ‘yes’ and to the ‘no’ answers, respectively).

### (d) Electroencephalography set-up

A total of 64 active electrodes connected to a BrainVision actiCHamp amplifier were placed according to the 10-10 montage in an actiCAP electrode cap except for four electrodes (FT9, FT10, TP9 and TP10) that were used as external electrodes. Two were placed around the eye to capture horizontal (HEOG) and vertical (VEOG) eye movements, and two were placed on the mastoids for offline re-referencing (M1, M2). During the recording, M1 was the online reference and FPz was used as the ground. The EEG signal was sampled at 1000 Hz and electrode impedances were kept below 10 KΩ.

### (e) Pre-processing of electroencephalography data

Pre-processing of the EEG data was done using Fieldtrip [39] on MATLAB R2022a [40]. The continuous EEG data was filtered with a line noise filter at 50 Hz, and a high pass filter at 0.5 Hz and a low pass filter at 50 Hz. Data were re-referenced from M1 to the average of both mastoids, and then segmented into 2.5 s trials (from −1 to 1.5 s with respect to stimulus onset). Trials in which the participant did not answer were discarded. Independent component analysis (*runica* method from Fieldtrip) was used to remove blinks and other possible artefacts, such as lateral eye movements or the heartbeat, if present (1.7 artefacts were removed on average per participant). Remaining artefacts were removed by visual inspection, discarding the trials (8.37 audiovisual trials were removed on average per participant in the pre-registered analyses). Two noisy data channels, from different participants, were interpolated through triangulation (with the *ft_channelrepair* function from the Fieldtrip package).

### (f) Paradigm validation

To check that the paradigm was working as expected, we performed three analyses on the behavioural data of both experiments. First, we expected the strongest ventriloquist effect at smaller disparities, and a decline thereafter. Auditory bias was calculated, as in previous literature [2,41], as the difference between the perceived and the real location of the sound divided by the actual spatial disparity (expressed as percentage). Second, we expected unity perception to peak at zero disparity and decline as disparity increased. The third validation analysis consisted of the estimation of the auditory localization bias separately for trials with and without perceived unity. We expected the auditory bias to be much stronger in the former, compared with the latter (following from [42], see also [2]).

### (g) Pre-registered behavioural analyses

For the RT calculation, we excluded trials more than 2.5 s.d. from the individual means. Trials in which the participant located the auditory stimulus at the same position of the visual stimulus but then answered that they were not coming from the same location were also excluded, as these trials were interpreted as errors (approx. 1.5% of the trials).

Two analyses were pre-registered. The first analysis addressed RTs as a function of audiovisual disparity. The initially pre-registered analysis grouped trials in three disparity categories: no disparity (i.e. spatially congruent stimuli), intermediate disparities (6° and 12°) and large disparities (18° and 24°). As we had enough power, and for consistency with the following EEG analyses, the main results section reports the RT across all disparities: 0° (100 trials), 6° (160), 12° (120), 18° (80), 24° (40). The results for the pre-registered categorical conditions can be found in the electronic supplementary material, figure S1. The mean RT for each disparity and participant was entered in a repeated measures ANOVA, followed by pairwise comparisons (Bonferroni corrected). In the second analysis, trials in which the participant reported that both stimuli were coming from the same position were split into congruent trials (0° of disparity) and incongruent trials (non-zero disparity). Although participants reported the same unity perception in both cases, we expected slower RTs for incongruent trials compared to the congruent ones. The mean for each condition was calculated for each participant and a one-tailed paired *t*-test was conducted to test this prediction.

### (h) Pre-registered electroencephalography analyses

As we did not have an *a priori* idea of the time course of the theta power (as it is strongly dependent on the paradigm, stimuli, and other methodological aspects), we pre-registered two large post-stimulus time windows for initial analyses: 0 to 0.5 s, and 0.5 to 1 s. The initial data analyses showed that the mean theta power increases (regardless of condition) in the earlier time window (see the electronic supplementary material, figure S3, and also figure 4 in the Results section). Therefore, from this point forward, our hypothesis testing, reported in the results sections, was run on this first time window (analyses for the second window can be found in the electronic supplementary material, figure S4).

For each participant, we measured theta power (5–7 Hz) difference at fm electrodes (Cz, FCz, Fz, FC1 and FC2) in the 0.5 s post-stimulus window, with respect to the baseline (−0.5 to 0 s) for each trial. Short-time Fourier transforms with a Hanning taper were used to calculate both theta powers (baseline and first window). To test our first hypothesis, the data was split into two categories: spatially congruent (100 trials) and incongruent trials (non-zero disparity, 400 trials), and compared with a one-tailed paired *t*-test (theta incongruent > theta congruent). Also, we estimated time-resolved theta power using short-time Fourier transform for the same electrodes (500 ms sliding windows), comparing the two conditions, correcting for the baseline (−0.75 to −0.25 s, to avoid including post-stimulus activity) mean power. We used one-tailed paired *t*-tests with a Monte Carlo approach to correct the significant cluster for multiple comparisons throughout the time points of the window (10 000 iterations). To test our second prediction, we entered theta power (baselined) at the fm cluster in a repeated measures ANOVA with disparity, followed with the pairwise comparisons (Bonferroni corrected).

### (i) Time-frequency and theta topography plots

To explore the spectral specificity of the possible effect we explored the contrast in the cluster of interest (Cz, FCz, Fz, FC1 and FC2) time-frequency analysis exploring frequencies from 2 to 30 Hz (in steps of 0.5 Hz). Short-time Fourier transforms with a Hanning taper with 500 ms sliding windows were used. Baseline correction was done subtracting the mean power from the baseline (−0.75 to −0.25), as in the previous time-resolved analyses. A topography of theta (5–7 Hz) was estimated at the peak of theta activity in the average time-frequency plot.

### (j) Theta source localization

The analysis was conducted using Fieldtrip, with the standard 10–20 electrode placement for the coordinates of the electrodes and a standard MRI template for the head model. The leadfield was calculated through the Fieldtrip *ft_prepare_leadfield* function setting a 5 mm grid resolution. For each participant, data was re-referenced to the average of all the electrodes, and the time window between 0 and 0.5 s was selected. The common filter for both conditions (congruent and incongruent trials) was computed with the 'dynamic imaging of coherent sources' method [43] and a regularization of 0.001% (low regularization set to avoid overlap). The common filter was then applied to both conditions to obtain the power estimates and the difference between the incongruent and congruent conditions was done. One-tailed paired *t*-tests were used to assess the significance of the clusters, and a Monte Carlo approach was used to correct for multiple comparisons (10 000 iterations).

### (k) Linear mixed modelling

To better understand the relationship between intersensory disparity and the behavioural and electrophysiological measures, linear mixed models were fitted to the data. In both models, we included disparity (factor), unity (factor) and location response (continuous variable) as fixed variables and participants as a random effect. Because participants answered with the left hand to the location of stimuli, but stimuli could come from left or right of the midline, we included location response as a variable in the model to account (and discount) for a possible Simon effect. We built different models for the RT and the EEG data using the *lmerTest* package [44] in R 4.2.1 [45]. Please note that in the results sections we applied the models to the same sample of participants included in the pre-registered analyses, but we later extended the models to the entire dataset of all participants (including the participants that had been excluded based on the pre-registered criteria) to show that the results were robust, and not affected by the predefined inclusion/exclusion criteria (see the electronic supplementary material, figure S7).

## 3. Results

In both experiments, the 200 unisensory trials were used to assess the exclusion criteria (55 out of 73, and 49 out of 59, passed the criteria for the RT and EEG experiments, respectively), and the 500 audiovisual trials were used to test the hypothesis. In these audiovisual trials, participants gave an initial sound location response and subsequently a unity response (yes/no answer). The main difference between the RT and the EEG experiment was that in the former, participants were asked to answer as fast as possible to the location of the sound, while in the latter participants had to wait for 1 s before answering. Before starting with the hypothesis testing, we wanted to confirm that the protocol worked as expected, based on the results of previous studies [2,41,42,46].

### (a) Paradigm validation

**Figure 2.**
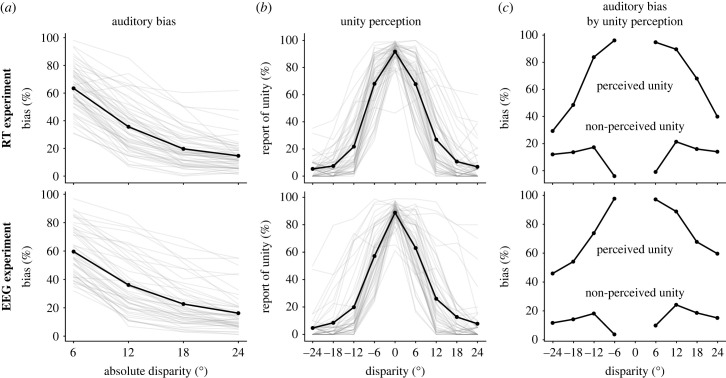
Paradigm validation analyses. The upper row corresponds to the RT experiment and the lower row to the EEG experiment. (*a*) Mean auditory bias (i.e. the influence the light has on the perceived position of the sound; black line) as a function of absolute disparity. Individual participant results shown with light grey lines. (*b*) Mean unity perception (i.e. the percentage of trials where both stimuli were perceived as coming from the same location; black line). Individual participant results shown as light grey lines. (*c*) Mean auditory bias as a function of relative disparity, distinguished by unity perception.

The validation analyses were run to ensure that the paradigm had the expected outcomes in sound localization and unity responses. The first validation analysis confirmed that auditory bias decreases with increasing disparity (see figure 2*a*). We observed average biases from 65% to 20%, at disparities from 6° to 24°. This goes in line with previous studies, such as Bertelson *et al*. [41] (drops from 57% to 32.6%, at disparities from 7° to 25°), and more recently, Körding *et al*. [2] whose protocol we adapted, who registered biases from 38% to 25%, using disparities ranging from 5° to 20°. The second validation analysis corresponds to the explicit unity judgements. The percentage of unity peaked at 0° disparity (mean unity responses of about 90%), and declined towards zero values with the increasing disparities (see figure 2*b*); similar to what has been obtained in other studies, such as Bertelson *et al*. [41], where the unity percentage drops from 79% to 12%, at disparities from 7° to 25°, or Körding *et al*. [2] using data from Wallace *et al*. [42], with unity judgements decreasing from 80% to 40% at disparities from 5° to 15°. The last validation consisted of assessing auditory bias by disparity separately for unity and non-unity perceived trials (see figure 2*c*). As expected, for unity trials ventriloquism was strong, while for non-unity events bias was weak (or even negative) meaning that perceived sound location was closer to its veridical origin (or even repulsed from the light) when no unity was experienced. This is similar to the pattern presented in Wallace *et al*. [42], reanalysed by Körding *et al.* [2]. Overall, we found the expected effects in the localization of the sound and unity perception, replicating previous results [2,36,41,42,46] and validating the paradigm to test our hypotheses.

### (b) Pre-registered response time analyses

**Figure 3.**
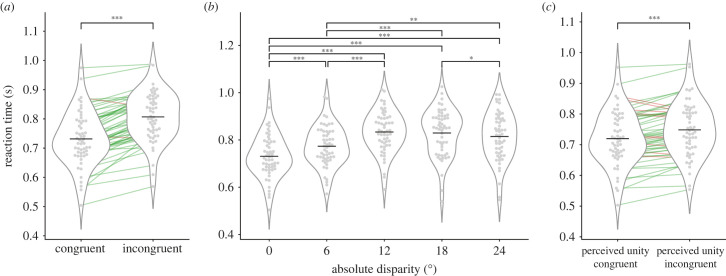
RT results. In all plots, grey dots correspond to individual participants' means and the horizontal black line corresponds to the group mean. Green and red lines unite individual data points, with colours indicating those participants whose pattern conforms to the initial prediction (green) and those that do not (red). (*a*) Mean RT for congruent (0° of disparity) and incongruent (disparity different from zero) trials. (*b*) Mean RT as a function of absolute disparities: 0°, 6°, 12°, 18° and 24°. (*c*) Mean RT for unity perceived trials as a function of whether they were physically congruent or incongruent. Significance levels: *p < 0.05; **p < 0.01; ***p < 0.001.

First, we analysed and found a significant 76 ms RT difference between congruent and incongruent trials (731 s.d. = ± 91 ms; versus 807 ± 84 ms; *t*_54_ = 12.926, one-tailed, *α* = 0.05, *p* = 1.83 × 10^−18^, Cohen's *d* = 1.74; figure 3*a*; see also the electronic supplementary material, figure S2 for the fitted gamma distributions for both conditions). For 53 out of 55 participants there was a numerical increase in the expected direction. We then entered the mean RTs in an ANOVA with the five disparity levels: 0°, 6°, 12°, 18° and 24° (figure 3*b*). The repeated measures ANOVA was significant (*F*_2.1,113.39_ = 62.82, *α* = 0.05, *p* = 1.22 × 10^−19^, *η*^2^ = 0.150, *η*_p_^2^ = 0.538), and all the pairwise comparisons between conditions were significant (Bonferroni corrected) except for the comparisons 12°–18° and 12°–24°. Please note that zero disparity trials were responded the fastest (confirming the first analysis), and overall, the pattern was, as predicted by the hypothesis, nonlinear: RTs increased from 6° to 12°, and then decreased from 18° to 24°. However, to better understand the relationship between RT and spatial disparity, we modelled the RT separating for other potential sources of variance (the results are presented below, in §3e *Linear mixed models*).

Second, we analysed RTs in the unity perceived trials separately, comparing those that were veridical congruent (0° disparity), 719 ± 87 ms, with actually incongruent trials, 748 ± 87 ms (figure 3*c*). The difference was significant (*t*_54_ = 6.923, one-tailed, *α* = 0.05, *p* = 2.743 × 10^−9^, Cohen's *d* = 0.934), and the difference was true for 45 out of the 55 participants. Therefore, despite participants experiencing ventriloquism and subjectively perceiving both types of events identically in terms of their causal origin, the processing time to estimate sound location in the incongruent trials was longer.

In accordance with the initial hypothesis, our results suggest that more processing time is needed to resolve the causal inference problem in incongruent trials, as it involves a steeper competition between the internal models (figure 3*a*). What is more, this increase in RT is more pronounced at 12° and 18° disparities and declines at 24°, where competition should be less steep (figure 3*b*).

### (c) Pre-registered electroencephalography analyses

**Figure 4.**
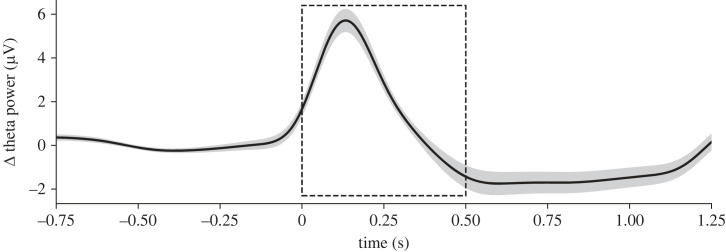
Mean time-resolved theta power across all trials. Theta power is baseline corrected subtracting the mean power between −0.75 and −0.25 for all data points. The dashed box corresponds to the analysed time window of interest throughout the results section.

In the EEG experiment the protocol included a one second delay before localization responses, to prevent motor contamination in the time window of interest for the EEG analysis. After seeing, that independently of congruency, the overall increases in theta power occurred from 0 to 0.5 s (figure 4; also electronic supplementary material, figure S3 for the plot distinguishing for the different disparities), we concentrated our analyses on this window (the results of the analyses for the second time window can be found in the electronic supplementary material, figure S4). The delta (Δ) power values correspond to post-stimulus theta power with respect to the baseline.

**Figure 5.**
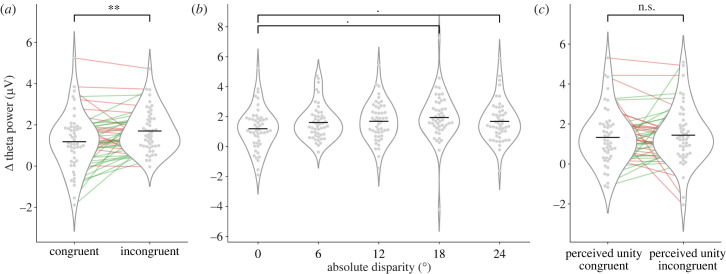
Δ Theta power results. Δ theta power (difference between the mean theta power in the window of interest and pre-stimulus baseline). In all plots, grey dots correspond to individual participants’ means and the horizontal black line corresponds to the group mean. Green and red lines unite individual data points, with colours indicating those participants whose pattern conforms to the initial prediction (green) and those that do not (red). (*a*) Mean Δ theta power for congruent and incongruent trials. (*b*) Mean Δ theta power as a function of disparity. The dots indicate the comparisons with a *p*-value < 0.1. (*c*) Mean Δ theta power for unity perceived trials which were physically congruent (disparity = 0) compared to unity perceived trials which were physically incongruent (non-zero disparity). Significance levels: *p < 0.05; **p < 0.01; n.s., not significant.

The first analysis tested for and found a significantly larger Δ theta power in incongruent (non-zero disparity) trials than in congruent ones (see figure 5*a*) (1.178 ± 1.387 µV versus 1.709 ± 1.032 µV; *t*_48_ = 3.027, one-tailed, *α* = 0.05, *p* = 0.00198, Cohen's *d* = 0.43). This pattern was true for 31 out of 49 participants, and the overall group effect conforms to the expected outcome.

The second analysis addressed Δ theta power across the five disparities (figure 5*b*). The repeated measures ANOVA was significant (*F*_3.09, 148.48_ = 3.43, *α* = 0.05, *p* = 0.018, *η*^2^ = 0.033, *η*_p_^2^ = 0.0668) but none of the pairwise comparisons turned out significant after correcting by Bonferroni, despite two being close to it: theta for 0° disparity (i.e. congruent stimuli) trials being lower than theta for 18° and 24° disparities (*p* = 0.055 and *p* = 0.093, respectively). Although the pattern of theta power follows, at least numerically, a nonlinear trend with larger values at intermediate disparities and a slight decline towards the largest disparities (similar to that of RTs), this trend was not confirmed statistically.

Finally, we analysed if there were differences in trials perceived as unity between physically congruent (0° disparity) and incongruent (non-zero disparity) trials (figure 5*c*). The difference was not significant (*t*_48_ = 0.484, one-tailed, *α* = 0.05, *p* = 0.315, Cohen's *d* = 0.07). This outcome, in principle, does not validate our prediction.

### (d) Characterization of the theta modulation

**Figure 6.**
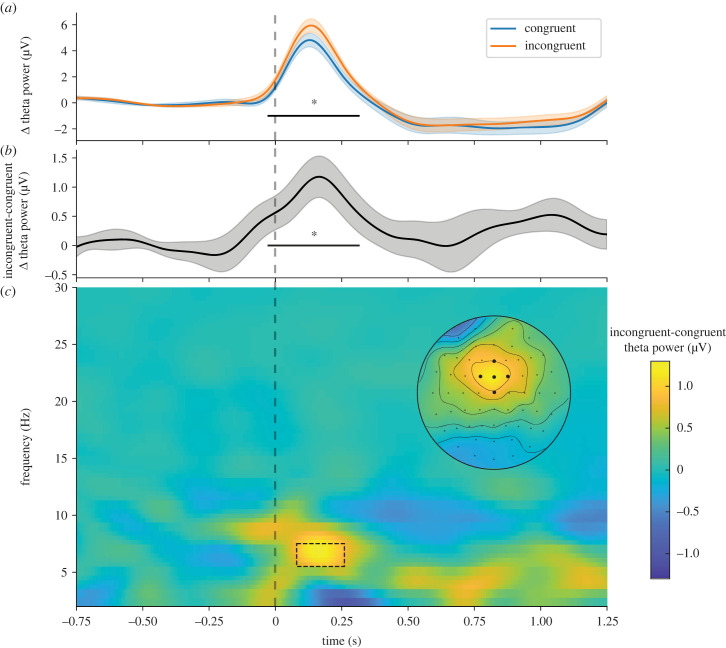
Time-resolved analyses. (*a*) Time-resolved theta power for the congruent (blue) and incongruent (orange) trials. Error shade corresponds to the standard error. The significant cluster was corrected by a Monte Carlo approach. (*b*) Difference between the incongruent and congruent theta power. (*c*) Short-time Fourier transforms with 500 ms sliding windows were used for the time-frequency analysis for the incongruent minus congruent comparison. The topography plot corresponds to the time-frequency window indicated by the dashed box. Significance level: *p < 0.05.

**Figure 7.**
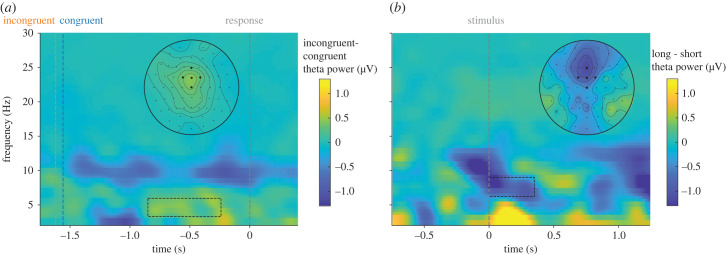
Time-frequency analyses. (*a*) Response-locked time frequency analysis without baseline correction. As seen, most of the theta activity seen right after stimulus onset disappears. In the dashed box, the main increment in theta power is observed. Visually compared to the stimulus-locked, the increment is at lower frequencies and located at posterior regions. (*b*) Stimulus-locked time frequency analysis with baseline correction (−0.75 to −0.25) for the difference between long congruent trials and short congruent trials. Visually, the theta power does not seem to increase in long compared to short trials (in fact, if anything, it would be the other way around).

The time-resolved analysis comparing theta power between congruent and incongruent trials (see figure 6*a,b*) returned significant differences in the studied window (−0.026 to 0.314 s, corrected by Monte Carlo with 10 000 iterations; *n* = 341, *t* = 858.98, *p* = 0.0250). The time-frequency plot (see figure 6*c*) suggests that the increase was specific of the theta band (5–7 Hz), and that theta originated from the fm electrodes, as can be seen in the topography plot (see figure 6*c***,** inset). Overall, these analyses indicate that theta power (and the difference between conditions) as a spatial and spectral profile is equivalent to the effects observed in classical motor conflict paradigms [14,16,18]. However, unlike the typical temporal profile observed in motor conflict, the theta modulation in this study occurred far from the response, and therefore, we assume, it must have been caused by a perceptual rather than motor conflict. To confirm this, we ran a response-locked time-frequency analysis and observed that most of the theta activity after the stimulus disappeared (figure 7*a*).

The ACC activity differences seen in incongruent trials when compared to congruent trials in fMRI studies had been debated, with the finding that the ACC activity increases with the time-in-trial [47,48]. As the mean incongruent RT is always higher than the mean congruent RT, the two effects (time-in-trial and incongruency) could not be disentangled. To rule out the possibility that the observed differences in our study were a consequence of an increased time-in-trial, we followed the same strategy as in Grinband *et al*. [47], and divided the congruent trials into short and long trials (the contrast is shown in figure 7*b*). The difference between long and short latency responses did not increase the theta power in our study (if anything, the trend is towards a decrease).

**Figure 8.**
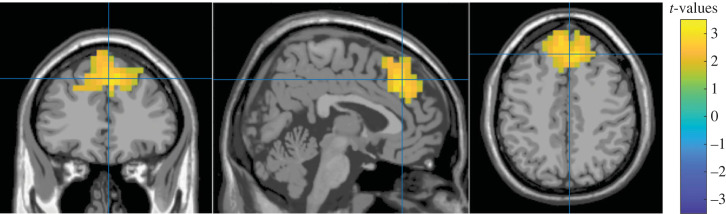
Theta source localization. From left to right: coronal, sagittal and horizontal views for the source localization of the theta power activity (5–7 Hz) in the first window (from 0 to 0.5 s) for the difference between incongruent and congruent trials is shown. Only the shown cluster was significant after the Monte Carlo correction.

To better characterize the observed modulation, we conducted a source localization analysis of the increase in theta power in the 0 to 0.5 s window, for the difference between incongruent and congruent trials. A significant cluster of activation after correction was found (10 000 iterations, *t* = 674.298, *p* = 0.0368) above the dorsal ACC (centred at the superior frontal gyrus) (figure 8), similar to the observed activation for incongruent versus congruent trials in an fMRI study with the classical motor conflict paradigms [49]. Given the use of standard MRI models and the sparse electrode montage, source localization cannot be very precise, but the source is generally consistent with the expected location.

### (e) Linear mixed models

**Figure 9.**
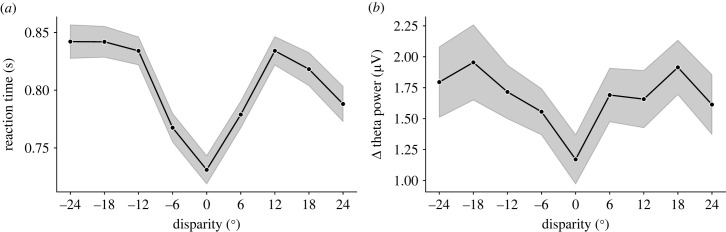
(*a*) Mean RT and (*b*) Δ theta power as a function of relative disparity. In both plots, the shade corresponds to the standard error of the means.

The main objective of the two experiments presented in this study was to gauge RT and theta power variations as a function of the spatial disparity between sound and light, and eventually the ventriloquist illusion (displacement of the perceived sound location). Our expectation was to find an increase for intermediate disparities and a decrease at higher disparities for the behavioural RT and neural correlates, owing to the hypothesis that causal inference would engage in steeper causal model competition at intermediate disparities, and therefore draw on the conflict brain network. These predictions were only partially confirmed in the pre-registered analysis above. Below, we open the analytical pipeline for a more extensive, exploratory approach. To start with, we distinguished between positive and negative disparities (negative disparities refer to the light coming from the left of the sound), instead of the absolute disparities used before. Figure 9 displays the mean RT (from the RT experiment) and theta power (from the EEG experiment) for each disparity (the RT from the EEG experiment followed a similar pattern to the RT observed in the RT experiment and can be found in the electronic supplementary material, figure S5). This already revealed a previously unforeseen source of variability. Although in principle, we assumed that left and right stimuli would be equivalent (hence, we collapsed this variable in the analyses), the left −18° and −24° disparities produced slower RTs and higher theta than the corresponding right 18° and 24° disparities. We argue that because participants responded to the location of the sound with their left hand, whenever the sound was presented to the right of the midline it may have led to a Simon-like effect and produced a spatial conflict of a sensory-motor origin (see [50–52]). To better study that, we averaged the RT for each participant at each finger for the unimodal trials. An increase in RT could be found from left to right fingers (see the electronic supplementary material, figure S6). To account for this response compatibility effect, as well as differences in the finger used to respond or other sources of variability such as faster responses in unity perceived trials, we decided to use linear mixed models (LMM) to conduct a more exhaustive analysis. In particular, we fitted separate LMMs with all disparities, perceived sound location (response finger), and perceived unity, as fixed effects, and participant as a random effect. For each of the two experiments (RT and EEG), we fitted the LMM to the participants' dataset used in the analysis (those that passed the pre-registered inclusion criteria) and then again including all participants’ datasets, to check the robustness of the outcomes (the latter analyses can be found in the electronic supplementary material, figure S7).

**Figure 10.**
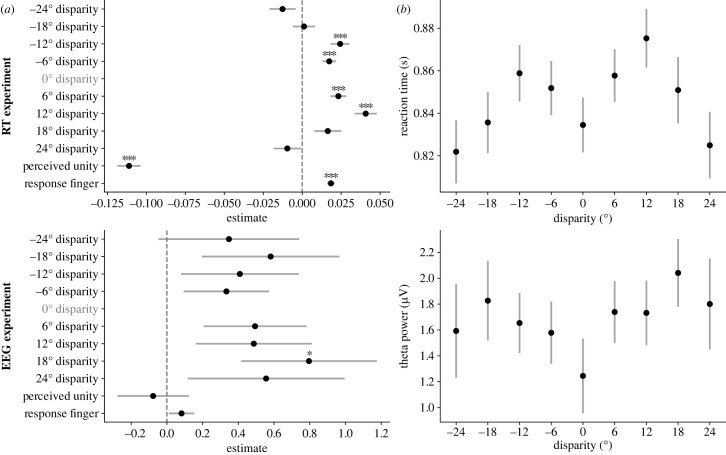
Linear mixed models results. The upper row corresponds to the RT experiment and the lower row to the EEG experiment. In both experiments, an LMM was fitted for the participants that passed the pre-registered criteria. Error bars correspond to the standard error. (*a*) Estimates of the fixed effects included in the model. The dotted line is the zero effect reference. (*b*) Model predicted values for each disparity, compensating for the effects of unity perception and perceived location (response finger). Significance levels: *p < 0.05; ***p < 0.001.

For the RT model (figure 10, upper row), perceived unity and response finger had a significant contribution to the effects, confirming the suspicion that a Simon effect may have added variability. Importantly, we found a nonlinear effect of disparity: disparities between −12° and 12° led to significantly slower RTs compared to the 0° disparity, whereas larger disparities rendered RTs not different from the 0° disparity. The LMM model predicted that RTs (accounting for the unity and location perceptions) increased from zero to 12° and −12° disparities and decreased at larger disparities. For the LMM fitted to theta power (figure 10, lower row), the variability was substantially larger. Neither response finger nor perceived unity had a significant effect on theta, and only the 18° disparity had an effect significantly different from 0° disparity (despite the overall theta being numerically higher than baseline). Despite that, the predicted values for the model (accounting for the unity and location perceptions) had a similar shape to the RT, with increased theta at smaller disparities which declined towards larger ones.

Overall, in the RT and, only to some extent in the theta power, the results were in line with our hypothesis. There was an increase in intermediate disparities (compared to no disparity) and a decrease in higher disparities, confirming the nonlinear relationship of these variables with disparity.

## 4. Discussion

We have addressed the hypothesis that the resolution of intersensory conflict, leading to the ventriloquist illusion and profusely used to model the multisensory perception process in general, draws from conflict monitoring and resolution brain mechanisms. Based on this hypothesis, we tested the prediction that RTs and fm-theta power responses induced by intersensory conflict increase upon the uncertainty and competition regarding the causal models that best explain the sensory event. Despite the abundant literature on the ventriloquist illusion [53] as a paradigm model to study multisensory processing, some aspects continue to be unexplored. First, to our surprise (and to the best of our knowledge), only one study has measured speeded RTs in a ventriloquism protocol [36]. Even so, this earlier study did not include gradual variations in intersensory disparity. Second, despite the ventriloquist illusion, and most multisensory illusions, are intrinsically based on intersensory conflict, multisensory literature has rarely reached out to the well-known conflict monitoring theory. For that matter, conflict literature has rarely addressed sensory conflicts either. Most of the literature on conflict monitoring focuses on motor conflict (such as the Stroop [14,15], the Flanker [16] or the Simon [17,18] tasks), and just a few studies recently started to test whether similar conflict responses are observed when the mismatch is produced at stimulus level [22,23,33].

With this in mind, we designed the present study to explore two typical responses to conflict (increases in RTs and in fm-theta power) in a ventriloquist paradigm, bridging the gap between these two domains of research. Inspired by the causal inference framework in multisensory perception, we hypothesized that the two alternative generative causal models regarding the sensory cues (common cause versus different causes) may engage in a competition process that would lead to the recruitment of the conflict network. So far, the data has validated some of the predictions of this hypothesis, as we found slower RTs and larger fm-theta to incongruent trials and higher RTs at intermediate disparities, compared to congruent and higher disparities. We also found the expected trend for fm-theta, but the differences between disparities were not significant, possibly owing to very low signal to noise ratio.

Our behavioural results are in accordance with the only speeded ventriloquist study that we could find, that reported faster responses to congruent compared to incongruent stimuli [36]. Another multisensory study also measured speeded RTs with different degrees of disparity between modalities, though looking at rate perception instead of spatial localization [54]. In this study, the authors observed a similar pattern to the one we obtained, at least when the auditory signal was reliable and task-relevant (and the visual signal irrelevant). Whenever there was no disparity between the visual and auditory rate, or the disparity was high, participant's responses were faster than at intermediate disparities. This outcome can be readily accommodated under the proposed hypothesis.

In the present study, the EEG results regarding stimulus induced theta power are generally analogous to the RT profile, though they were not as clear cut, in part owing to the intrinsic noise of the measure. Please note that RTs and theta were measured in different studies with slightly different protocols to protect from the trivial interpretation that theta power and RTs are often correlated in perceptual decision-making tasks and cannot be disentangled (but see [55]). Here, theta power was estimated in a fixed delay period between the stimulus and the prompt for response.

Under these conditions, we observed a robust significant increase in theta power after incongruent compared to congruent trials (figure 5*a*), that was specific for the theta band (5–7 Hz) (figure 6*c*) and originated from a fronto-central electrode cluster (figure 6*c*, inset); therefore, reflecting similar spectral and spatial properties to the theta increases observed in classical motor conflict paradigms. Regarding the temporal course, theta power peaked within 250 ms after the stimulus, similar to the observed peak latency for cross-modal conflict previously observed using the McGurk illusion [22]. Despite a clear increase in theta power after stimulus presentation, and that this increase was significantly larger for disparity trials compared to congruent ones, not many significant differences were found for the theta power across the non-zero disparities. However, the numerical pattern of theta power across disparities was as expected by our hypothesis, with an increase in intermediate disparities, and a decrease at larger ones. Please note that in any case, theta did not simply correlate with disparity.

According to various proposals regarding the hierarchical implementation of causal inference in the brain, multisensory perception would begin with the computation of unisensory estimates in sensory areas [54,56,57] during the first 150 ms [54,58], followed by a fused estimate in posterior association brain regions [54,57], 100 ms to 260 ms [54,58], and finally the computation of the causal inference estimate that would involve anterior regions starting as soon as 200–300 ms [59] (timings vary between studies [54,58]). The observed peak of theta in our study would fall between the timing of the generation of the fused estimate and the generation of the causal inference estimate. This timing fits well with the idea that the origin of the conflict signal is the arbitration between internal causal models, prior to the emergence of the final multisensory estimate. Because of this timing profile, and the fact that fm-theta was not just proportional to disparity (despite the differences between disparities were not significant, the highest theta power was found at the intermediate disparities), we argue that theta might reflect the competition between the causal models, occurring right before the perceptual decision that best explains the sensory event.

Previous studies addressing theta power and/or ACC activity in multisensory paradigms generally achieved results compatible with the present ones and could be interpreted within our proposed intersensory conflict view. For example, an fMRI study with a ventriloquist paradigm with three disparities found an increase in ACC activity (as well as in the superior frontal gyrus, the anterior insula, the superior parietal lobules and other regions) for incongruent trials compared to congruent ones [25]. The authors concluded that these regions were activated as a consequence of the detection of the spatially incongruent stimuli. We propose another interpretation where the activation is not directly caused by the disparity between the stimuli, rather as a result of the competition between the two possible causal models. It would be interesting to see if the activation they observed would be diminished at higher disparities between the stimuli, as happened with the RTs and, at least as a trend, with the theta power in our study. The only study, to our knowledge, that finds ACC activity that would be incompatible with our proposed interpretation is an fMRI study with cross-modal semantic conflict, where they found an increase in ACC activity for semantically congruent pairs of images and sounds compared to incongruent ones [27]. As mentioned in the introduction, there are large methodological differences between this study and the present one. For example, they blocked congruent (or incongruent) pairings, thereby possibly leading to adaptation effects. To our knowledge, this outcome has not been replicated by other cross-modal semantic congruence fMRI studies (see [28,29] for reviews).

Other studies have also investigated theta power in other aspects of multisensory processing. For example, an EEG ventriloquism study found lower pre-stimulus theta power in trials in which participants experienced the illusion versus trials in which they did not [60]. This result does not necessarily go in opposition to ours, as it explored the pre-stimulus period, instead of post-stimulus induced activity. Their interpretation of the results was that a lower theta power reflects a lower cognitive control state that leads to a higher cross-modal influence.

We would like to point out some limitations of the current study. One such limitation relates to the potential role of conflict at the response level, as in our study participants reported their perception by key press (a debate parallel to concerns of the role of response selection in binocular rivalry protocols, see [61–64]). Despite the EEG experiment incorporating a response delay, and our response-locked analyses (figure 7*a*) did not show any sign of conflict, this still remains a logical possibility. This question could be better answered in the future with the design of no-report paradigms. Another limitation concerns alternatives to our account. One such alternative would be an active (rather than passive) inference account of perception, along the lines recently proposed by [65]. In these accounts, not only are the noisy perceptual estimates considered, but also the utility of the decisions (considering the benefits and/or costs of the outcome), for the final perceptual decision. Under this framework, multisensory objects might carry some intrinsic value (some sort of associated pleasure, for example) [66], difficult to account in our study. How both the active inference and the intrinsic multisensory values can affect the current study is uncertain. Considering the simplicity of our study, with beeps and flashes, these aspects might not have an important role (as they might have in real-world environments, where multisensory objects carry meaning, affordances, and expectations). Despite that, these aspects could be considered for the design of future studies. Finally, another limitation to bear in mind for future studies (and perhaps for the interpretation of earlier ones) is the possible impact of a Simon-like effect in our study afforded by the mapping between stimulus location, response hand, and response keys. This effect forced us to work with the unaggregated disparities, reducing to half the number of trials per condition (in the LMMs) compared to the pre-registered analyses. Despite this was not a problem for the RT experiment, this may have made it difficult to find conclusive results for the theta modulation at the different disparities. Future studies using larger disparities, finer estimates of brain activity, and/or with more statistical power might be able to confirm or negate this prediction (though bear in mind that we used a sample size well-above what is normally used in ventriloquist studies).

In summary, the main objective of the present study was to address the possible role of the conflict monitoring system in multisensory integration. We proposed that the conflict mechanism would be activated owing to the competition between representations of alternative causal models that generated the stimuli (common cause versus different causes). So far, our results are consistent with this idea, suggesting that at intermediate disparities, where the putative competition between viable causal models (integration versus segregation) is the highest, we observed a significant RT slow down and a non-significant trend towards an increase in theta power. Future studies with higher number of participants, larger studied disparities or other intersensory conflict paradigms may help to further confirm this hypothesis.

## Data Availability

Code and data can be found from the OSF repository: https://osf.io/6yrwq/. The supplementary figures are provided in the electronic supplementary material [67].
